# Insight into cordycepin biosynthesis of *Cordyceps militaris*: Comparison between a liquid surface culture and a submerged culture through transcriptomic analysis

**DOI:** 10.1371/journal.pone.0187052

**Published:** 2017-11-01

**Authors:** Ahmad Suparmin, Tatsuya Kato, Hideo Dohra, Enoch Y. Park

**Affiliations:** 1 Laboratory of Biotechnology, Department of Agriculture, Graduate School of Integrated Science and Technology, Shizuoka University, Ohya, Suruga-ku, Shizuoka, Japan; 2 Laboratory of Biotechnology, Green Chemistry Research Division, Research Institute of Green Science and Technology, Shizuoka University, Ohya, Suruga-ku, Shizuoka, Japan; 3 Instrumental Research Support Office, Research Institute of Green Science and Technology, Shizuoka University, Ohya, Suruga-ku, Shizuoka, Japan; Universita degli Studi di Pisa, ITALY

## Abstract

*Cordyceps militaris* produces cordycepin, which is known to be a bioactive compound. Currently, cordycepin hyperproduction of *C*. *militaris* was carried out in a liquid surface culture because of its low productivity in a submerged culture, however the reason was not known. In this study, 4.92 g/L of cordycepin was produced at the 15^th^ day of *C*. *militaris* NBRC 103752 liquid surface culture, but only 1 mg/L was produced in the submerged culture. RNA-Seq was used to clarify the gene expression profiles of the cordycepin biosynthetic pathways of the submerged culture and the liquid surface culture. From this analysis, 1036 genes were shown to be upregulated and 557 genes were downregulated in the liquid surface culture compared with the submerged culture. Specifically, adenylosuccinate synthetase and phosphoribosylaminoimidazole-succinocarboxamide (SAICAR) synthase in purine nucleotide metabolism were significantly upregulated in the liquid surface culture. Thick mycelia formation in the liquid surface culture was found to induce the expression of hypoxia-related genes (GABA shunt, glutamate synthetase precursor, and succinate-semialdehyde dehydrogenase). Cytochrome P450 oxidoreductases containing heme were also found to be significantly enriched, suggesting that a hypoxic condition might be created in the liquid surface culture. These results suggest that hypoxic conditions are more suitable for cordycepin production in the liquid surface culture compared with the submerged culture. Our analysis paves the way for unraveling the cordycepin biosynthesis pathway and for improving cordycepin production in *C*. *militaris*.

## Introduction

Cordycepin is a bioactive compound that is produced by the genus *Cordyceps* and that has been used as a medicinal agent, including as an antimicrobial and an anticancer agent. There have been many attempts to produce cordycepin by chemical synthesis [1], extraction from *C*. *militaris* [2,3] and microbial fermentation of *C*. *militaris* [4,5]. Microbial fermentation in a liquid surface culture (LSC) or in a submerged culture (SMC) of *C*. *militaris* is a promising method for cordycepin production. To improve cordycepin production in SMC, dissolved oxygen was controlled at 60% at the beginning of the cultivation and reduced to 30% when cordycepin production started. However, cordycepin production only reached 0.2 g/L [6], indicating the difficulty of using the SMC for cordycepin production. Alternatively, cordycepin-hyperproducing *C*. *militaris* mutants were isolated by mutagenic technology using 8-azaadenine and proton beam irradiation [7], and these mutants produced 14.3 g/L of cordycepin under an optimized culture condition [8]. This type of successful hyperproduction of cordycepin has only been reported to occur in the LSC, but the reason for this has not yet been determined.

Transcriptomic analysis has been widely used to investigate the gene expression of microorganisms. This type of analysis provides information regarding gene expression patterns in different culture conditions, leading to new insights into genes that play critical roles in secondary metabolite production [9,10]. RNA-sequencing (RNA-Seq) is a prominent tool not only for obtaining sequence information but also for quantifying gene expression of several RNAs in targets [11,12]. Sequences mapped from the transcriptome can provide a reference genome for improving structural annotation, predicting alternative splicing and enlarging current databases. Zheng et al. reported a genomic analysis of the cordycepin producer *C*. *militaris* that provides information regarding the cordycepin biosynthetic pathway and signal transduction pathway that regulate fruiting body formation [13]. Moreover, analysis of differentially expressed genes was performed by transcriptomic and proteomic approaches in the mycelia and fruiting body of *C*. *militaris*. From this research, most genes that were involved in purine nucleotide metabolism were upregulated in mycelia compared with the fruiting body, suggesting that the cordycepin production pathway is much more active in the mycelia than in the fruiting body [14]. However, while considerable researches regarding the production of cordycepin in *C*. *militaris* has been conducted in the LSC, the difference in the cordycepin biosynthetic pathway between the LSC and the SMC has not yet been elucidated.

In this study, we analyzed gene expression differences in the biosynthesis of cordycepin between LSC and SMC using *C*. *militaris* NBRC 103752 and RNA-Seq. The results revealed the reason why the LSC is more suitable for cordycepin production than the SMC. Further, we identified specific genes that are upregulated in the LSC and speculated on the cordycepin production pathway of *C*. *militaris* NBRC 103752.

## Materials and methods

### Fungal strain

*C*. *militaris* NBRC 103752 was purchased from the Biological Research Center (NITE, Tokyo, Japan) and used throughout this research. The mycelia of the strain were dissolved in 1 ml of medium, which was composed of 5 g/L peptone, 3 g/L yeast extract, and 1 g/L MgSO_4_·7H_2_O. The suspension liquid was then transferred to potato dextrose agar (PDA) (Nissui Pharmaceutical Co. Ltd., Fuji, Japan) and incubated for at 25°C 7 d. Mycelia in PDA plates were used for cultivation in liquid medium, and the PDA slants were stored at 4°C as a stock culture.

### Medium preparation and culture conditions for cordycepin production

*C*. *militaris* was cultivated in an optimized production medium [15] composed of 72.5 g/L yeast extract, 62.6 g/L glucose (pH 5.6) and Vogel’s medium with 1/10 concentration containing 0.28 g/L sodium citrate dihydrate, 0.50 g/L KH_2_PO_4_, 0.20 g/L NH_4_NO_3_, 0.02 g/L MgSO_4_·H_2_O, 0.01 g/L CaCl_2_·2H_2_O, 0.46 × 10^−3^ g/L citric acid, 0.50 × 10^−3^ g/L ZnSO_4_, 0.1 × 10^−3^ g/L Fe(NH)_4_(SO_4_)_2_·6H_2_O, and 0.025 × 10^−3^ g/L CuSO_4_·5H_2_O. One disk (1 cm diameter) of mycelia in PDA plates that had been cultured for 7 d was inoculated into 100 ml of the optimized production medium in a 500 ml Erlenmeyer flask and incubated at 25°C for 19 d in the LSC. In the case of the SMC, *C*. *militaris* cells were cultured in a shaker with an agitation rate of 110 rpm at 25°C. The culture was sampled at the seventh day after inoculation and every two days after.

To investigate the effect of aspartic acid on cordycepin production, aspartic acid was sterilized using a 0.22 μm filter (Cat No. C020G047A, Advantec Co. Ltd., Tokyo, Japan) and added over a range of 0.5 to 1.5 g/L after the optimized production medium was sterilized.

### Quantitative analysis of cordycepin

Samples were thawed and centrifuged at 15,000×g at 4°C for 10 min. The supernatant was mixed with 2% methanol at a 1:1 ratio and filtered through a 0.45 μm filter (Cat No. HAWP04700, Millipore, Billerica, MA, USA) before analysis. The cordycepin concentration in the supernatant was measured by high-performance liquid chromatography (HPLC) with an UV detector at 260 nm (Shimadzu, Tokyo, Japan). The TSK-gel ODS-80Ts (Tosoh Corp., Tokyo, Japan) was used at 40°C with 0.1% phosphoric acid: methanol at a 98:2 ratio (v/v) was used as the mobile phase [8]. Cordycepin (Wako Pure Chem. Ind. Ltd., Osaka, Japan) was used as the reference standard.

### RNA isolation, cDNA preparation, and sequencing

*C*. *militaris* mycelia were grown in LSC and SMC for 19 d. The 13 d and 5 d, the exponential phase of cordycepin production in LSC and SMC, respectively, were chosen for RNA extraction and transcriptomic analysis. RNA was extracted from both cultures with Trizol reagent (Invitrogen, Carlsbad, CA) according to the manufacturer’s protocol. Briefly, 100 mg of mycelia from both cultures were measured in a 2 ml Eppendorf tube and homogenized in liquid nitrogen, and 1 mL of Trizol was directly added. The mixture was incubated for 5 min at room temperature; then, 200 μl of chloroform in 1 mL of Trizol was added, followed by centrifugation at 12,000×g for 15 min. The supernatant was removed, and the resulting pellet was washed with 1 ml of 75% ethanol. The pellet was then dissolved in 50 μl of nuclease-free water. Total RNA was treated using RNase-free DNase I (Qiagen, South San Francisco, Canada). Finally, RNA integrity was assessed using an Agilent 2100 Bioanalyzer (Agilent Technologies, Tokyo, Japan), and the quality was checked by agarose gel electrophoresis and a Nanodrop 2000c (Thermo Scientific, Wilmington, DE, USA). Total RNA samples (800 ng) extracted from the LSC and the SMC (n = 3 each) were used for strand-specific RNA-seq library construction by the KAPA Stranded mRNA-seq Kit (KAPA BIOSYSTEMS, Nippon Genteics Co. Ltd., Tokyo, Japan) according to the manufacturer’s protocol. These libraries were then sequenced (76-bp paired-end) by a MiSeq system (Illumina, San Diego, CA, USA) at the Instrumental Research Support Office of the Research Institute of Green Science and Technology at Shizuoka University.

### Raw data processing, de novo assembly and differential gene expression analysis

The raw reads were cleaned using Trimmomatic [16] to remove adapter sequences, bases with low quality (quality score <15), the last 76 bases, and reads of less than 50 bp. The cleaned reads were assembled de novo by the Trinity program version 2.1.1 with the option ‘—jaccard_clip’ to minimize fusion transcripts [17]. Ribosomal RNA sequences were excluded from the transcripts by removing sequences aligned to the SILVA rRNA database [18]. Differential gene expression analysis was performed by the scripts bundled in the Trinity package. Briefly, the cleaned reads were aligned to the Trinity-assembled transcripts using Bowtie [19], and then, transcript abundance was estimated using RNA-Seq by Expectation Maximization (RSEM) [20]. Differentially expressed genes between the LSC and the SMC were identified by the edgeR package [21]. To normalize for RNA composition by finding a set of scaling factors for the library sizes, we used the Trimmed Mean of M-values (TMM) normalization method [22]. The correlation of the transcript counts among replicates of the LSC and the SMC were assessed by a multi-dimensional scaling (MDS) plot and a cluster dendrogram [23]. Upregulated and downregulated genes were identified by the exact test in the edgeR package and defined by the log2-fold change, logFC ≥1 and logFC ≤-1, respectively, with a false discovery rate (FDR) cutoff of 5% (S1 Table). The edgeR script used for assessment of the transcript counts and identification of the differentially expressed genes was shown in the S1 Note.

### Gene Ontology (GO) enrichment analysis

The transcripts of the *C*. *militaris* NBRC 103752 were translated by the TransDecoder version 2.0.1 and annotated by hmmscan (http://hmmer.org/) against the Pfam database release 29.0 [24]. Pfam IDs were converted to Gene Ontology (GO) terms [25] using the pfam2go conversion table (http://geneontology.org/external2go/pfam2go). We performed parametric analysis of gene set enrichment (PAGE) [26] to define the enriched GO terms based on the logFC between the LSC and the SMC (S1 Table).

### Functional annotation

Functional annotation of the transcripts was analyzed by a local BLAST algorithm using the National Center for Biotechnology Information non-redundant (NCBInr) database (parameters: E-value: 1E-5, coverage >60%, identity >50%). Eukaryotic Orthologous Groups (KOG) of proteins was utilized to determine the functional classification of each gene based on the KOG database with an E-value of 1E-5 [27]. The protein sequences of the transcript were predicted by the Trans Decoder of the Trinity package. Further, the transcript was assigned to the Kyoto Encyclopedia of Genes and Genomes (KEGG) database (E-value cut-off 1E-10) by the GhostKOALA server (http://www.kegg.jp/ghostkoala/) [28]. A metabolic network was reconstructed using the KEGG mapper (http://www.genome.jp/kegg/) with KO numbers (S2 Table).

### Semi-quantitative RT-PCR verification

To validate the RNA-Seq results, expression of upregulated and downregulated genes of the LSC and the SMC were confirmed by semi-quantitative RT-PCR (qRT-PCR) using the primer set described in Table 1. The relative gene expression in an agarose gel was then quantified against the ubiquitin carrier protein [29] as a control by the Image J program (National Institutes of Health, Bethesda, Maryland, US).

**Table 1:** Primers for semi qRT-PCR.

Name	Primers (5' ⇒3')
Cm103752_Adenosine deaminase	F: CGACCAAGATGGAGTTCCCC
	R: CACTGCTTGCGGAACCAAAA
Cm103752_Adenylosuccinate- lyase	F: TCTGAGAGAGAGCTCGGCAT
	R: GTCGCACCGTAGTGGATGAT
Cm103752_Adenylosuccinate- synthetase	F: GAGATTGCCCGGTTCAAGGA
	R: TTGTAACGTGGGGGTACGTG
Cm103752_Ubiquitin carrier protein	F: ATGCTAGCCGCCAGCGTCCCC
	R: TTACAGCTCATCGCCGTTGTC
Cm103752_IMP cyclohydrolase	F: CTGCAGATTGACGCCGACTA
	R: TGCGTGTACTTGAGCGTGAT
Cm103752_SAICAR synthase	F: ACGGAGTCACGGGATTCTTG
	R: AAATGTCATGGAGCAGAGCT
Cm103752_5'-Nucleotidase	F: GTTCTCTCCGAGGCCCTAGA
	R: AAACCCGGACGCGATAAAGT

## Results

### Comparison of cordycepin production between the LSC and the SMC

**Fig 1:**
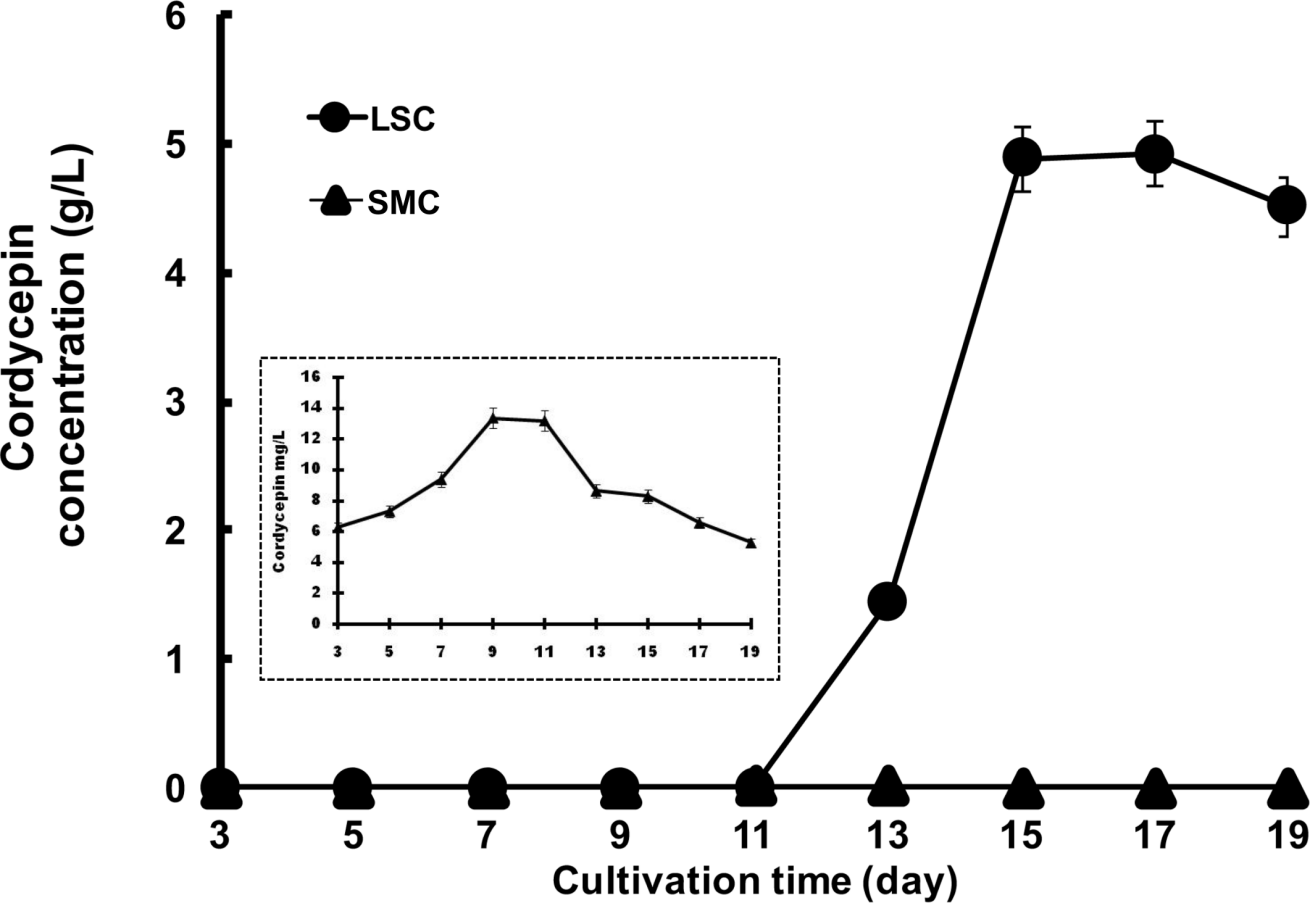
Cordycepin production in the LSC and the SMC of *C*. *militaris* NBRC103752. *C*. *militaris* cells were cultivated in the LSC and the SMC, and the concentration of cordycepin was measured every two days from the 3^rd^ d to the 19^th^ d of culture. Circles: LSC, triangles: SMC.

In the LSC, the cordycepin was produced in culture medium starting at 11^th^ d after inoculation and reached 4.92 g/L after 15 d (Fig 1). After the 15^th^ d of the culture, the amount of cordycepin was maintained in the culture broth until the 19^th^ d. By contrast, the cordycepin concentration in the SMC was 1 mg/L at 7 d and had decreased by the 19^th^ d of culture. These results suggested that the LSC is suitable for cordycepin production. Thick mycelia formed by *C*. *militaris* in the LSC covered the medium surface and seemed to create a hypoxic condition in the medium. By contrast, thin hypha and yeast formed in the SMC (S1 Fig).

### Sequencing and de novo assembly

To distinguish the transcriptome profiles during cordycepin biosynthesis, RNA-seq libraries were prepared from the total RNAs extracted from mycelia at the exponential phase of cordycepin production on the 13^th^ d and 5^th^ d of culture for the LSC and SMC, respectively. The RNA-seq libraries were then sequenced using Illumina MiSeq. After filtering, we obtained approximately 18.7 million (9.36 million pairs) and 19.6 million (9.8 million pairs) paired-end reads for the LSC and the SMC, respectively, containing a total of 2.86 Gb of nucleotides. All reads derived from both the LSC and the SMC were de novo assembled using Trinity, and then rRNA sequences and transcripts that were lowly expressed (read count ≤2) were excluded from the transcript contigs. Finally, we obtained 19,285 transcripts that were clustered into 17,620 unigenes. The transcripts had a maximum length of 15,412 bp, a median length of 1,154 bp, an average length of 1,623.55 bp, an N50 length of 2,683 bp, and a total length of 31,310,107 bp. To assess transcriptome completeness, the transcriptome was tested using BUSCO version 2.0.1 [30]. The result of BUSCO showed that the transcriptome covered 1,274 (96.9%) of the universal orthologs in Ascomycota, indicating the comprehensiveness of the transcriptome.

### Functional annotations of unigenes and differential gene expression analysis

The *C*. *militaris* unigenes were annotated by BLASTX with an E-value cut-off 1E-5 against the NCBInr databases and additionally annotated by a Pfam search. Of the 17,620 unigenes, 12,541 (71.2%) showed similarities to proteins in the NCBInr database and 8,037 (45.6%) were assigned to the Pfam protein families. To identify genes that were expressed differentially between the LSC and the SMC, we performed the transcriptome analysis by RNA-seq. Prior to identification of differentially expressed genes, we confirmed that the transcript counts for the replicates of the LSC and the SMC samples were correlated within samples (S2 Fig). The differentially expressed genes were detected by the exact test in the edgeR. Of the 17,620, 1593 were selected unigenes with logFC ≥1 or logFC ≤-1 with a FDR <0.05. The 1593 were expressed differentially between the LSC and the SMC: 1,036 were upregulated and 557 were downregulated in the LSC included hypothetical proteins S1 Table and S3 Fig, which produced a large amount of cordycepin.

### GO enrichment analysis of unigenes

To identify significant biological changes in the gene expression of the LSC and the SMC, we performed GO enrichment analysis by PAGE based on the logFC. PAGE detected enrichment of 19 GO terms, including 4 biological processes, 5 cellular components, and 10 molecular functions with FDR <0.05 (Table 2). The top 3 significantly upregulated GO terms in LSC were “heme binding” (GO:0020037), “iron ion binding” (GO:0005506), and “oxidoreductase activity, acting on paired donors, with incorporation or reduction of molecular oxygen” (GO:0016705). The top 3 downregulated GO terms in the LSC were “DNA binding” (GO:0003677), “structural constituent of ribosome” (GO:0003735), and “translation” (GO:0006412).

**Table 2:** GO term enrichment analysis between LSC and SMC of *C*. *militaris*.

GO_Name	GO_ID	Number of sequences	LogFC	Z-score	FDR
BP Transmembrane transport	GO:0055085	544	-0.4924	-4.2743	6.71×10^−6^
BP Proteolysis	GO:0006508	146	-0.7813	-4.0622	1.42×10^−3^
BP Transcription, DNA-templated	GO:0006351	194	-0.3013	-3.2980	1.22×10^−2^
CC Extracellular region	GO:0005576	16	-2.0209	-3.9689	1.58×10^−3^
CC Integral component of membrane	GO:0016021	581	-0.3739	-2.9064	3.64×10^−2^
CC intracellular	GO:0005622	94	-0.4239	-2.9254	3.64×10^−2^
CC Ribosome	GO:0005840	106	-0.4995	-3.5178	6.35×10^−3^
CC Nucleus	GO:0005634	432	-0.1544	-3.3062	1.22×10^−2^
MF Oxidoreductase activity*	GO:0016705	71	1.4280	5.7168	6.33×10^−7^
MFRNA-directed RNA polymerase activity	GO:0003968	11	-2.8440	-4.7357	9.55×10^−5^
MF Aspartic-type endopeptidase activity	GO:0004190	42	-1.1997	-3.6137	5.28×10^−3^
MF DNA binding	GO:0003677	332	-0.2656	-3.9705	1.58×10^−3^
MF Structural constituent of ribosome	GO:0003735	110	-0.5077	-3.6296	5.28×10^−3^
MF Sequence-specific DNA binding RNA polymerase II transcription factor activity	GO: 0000981	240	-0.2211	-3.0112	3.04×10^−2^
MF Translation	GO:0006412	109	-0.5008	-3.5748	5.58×10^−3^
MF ATPase activity	GO:0016887	131	0.6247	-2.8992	3.64×10^−2^
MF Iron ion binding	GO:0005506	87	1.3902	6.1416	7.15×10^−8^
MF Heme binding	GO:0020037	86	1.43846	6.3428	3.04×10^−2^
MF Serine-type endopetidase	GO:0004252	34	1.0610	2.8234	4.38×10^−2^

*Oxidoreductase activity, acting on paired donors, with the incorporation or reduction of molecular oxygen

BP: Biological process; CC: Cellular component; MF: Molecular function

### Difference in gene expression related to the cordycepin biosynthesis pathway between the LSC and the SMC

**Fig 2:**
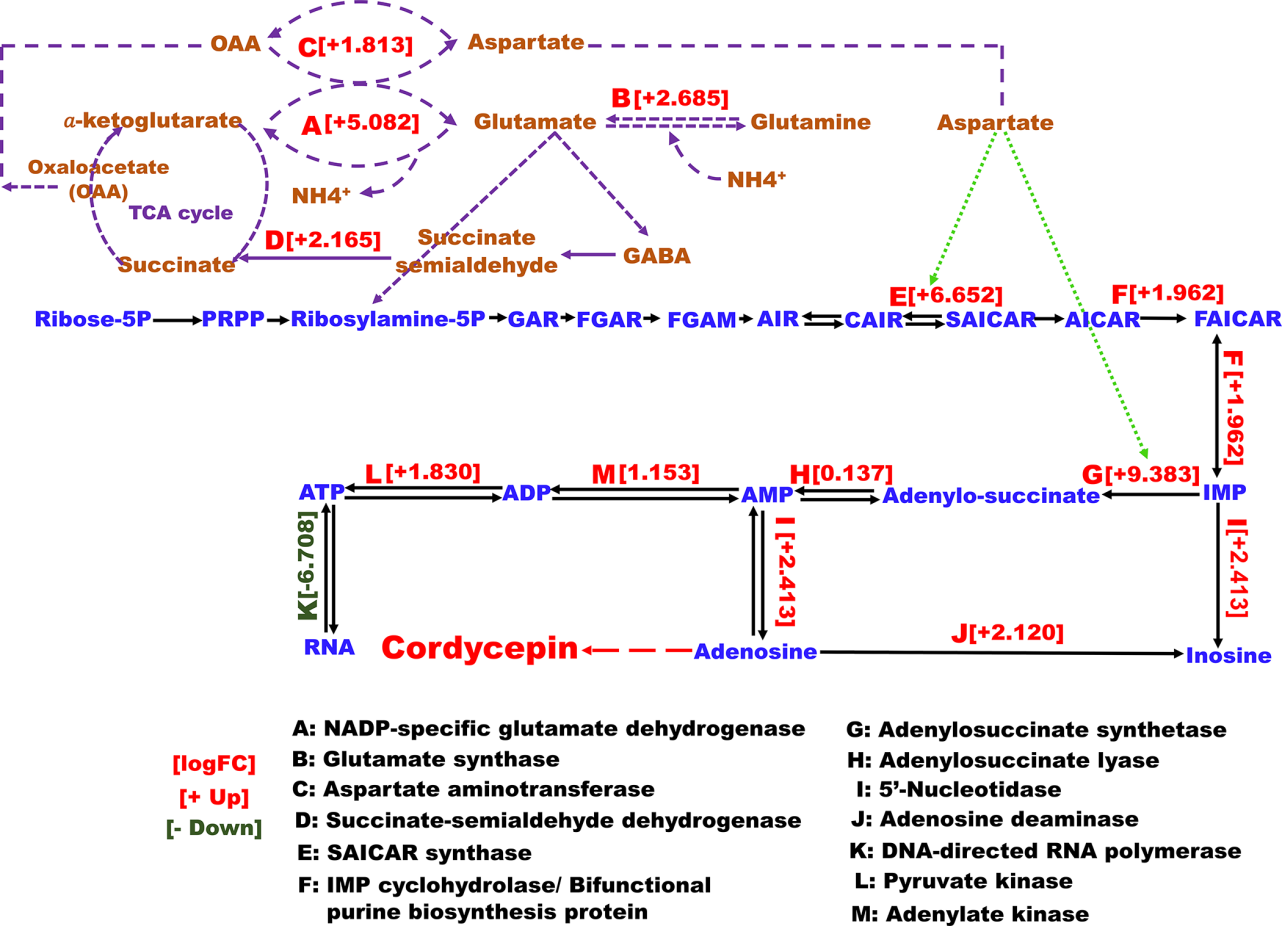
Differentially expressed enzymes of purine nucleotide metabolism in the LSC and the SMC of *C*. *militaris* NBRC103752. Numbers in parentheses indicate the upregulated or downregulated LogFC value. Round dotted, dashed, and long dashed lines denote precursors, different pathways, and proposed cordycepin biosynthesis, respectively. PRPP: phosphoribosyl pyrophosphate, GAR: glycinamide ribonucleotide, FGAR: phosphoribosyl-N-formyl glycine amide, FGAM: 5’-phosphoribosylformylglycinamidine, AIR: aminoimidazole ribonucleotide, GABA: **γ-**aminobutyric acid, AICAR: 5-Aminoimidazole-4-carboxamide ribonucleotide, AMP: adenosine monophosphate, Asp: aspartate, CAIR: 1-(5-phospho-D-ribosyl)-5-amino-4-imidazolecarboxlate, IMP: inosine monophosphate, SAICAR: phosphoribosyl aminoimidazole-succinocarboxamide.

The purine nucleotide is regarded as an important precursor for cordycepin production in *C*. *militaris*. Therefore, we focused on the difference in gene expression of the purine nucleotide biosynthesis pathway between the LSC and the SMC of *C*. *militaris*. Expression of phosphoribosyl-aminoimidazolesuccino-carboxamide (SAICAR) synthase (DN11023_c1_g1), phosphoribosyl aminoimidazole-carboxamide formyltransferase/IMP cyclohydrolase (bifunctional purine biosynthesis protein, DN11002_c0_g1), adenylosuccinate synthetase (DN10264_c0_g1), 5’-nucleotidase (DN1703_c0_g1), and adenosine deaminase family protein (DN3243_c1_g1) was significantly upregulated in the LSC compared with the SMC (Table 3). The expression levels of SAICAR synthase and adenylosuccinate synthetase genes were the most upregulated in the LSC, by logFC 6.65 (FDR: 9.88 × 10^−16^) and 9.38 (FDR: 8.64 × 10^−9^), respectively. SAICAR synthase (Fig 2E) transforms 5’-phosphoribosyl-4-carboxy-5-aminoimidazole (CAIR) to SAICAR, which is subsequently converted to inosine monophosphate (IMP). Adenylosuccinate synthetase (Fig 2G) acts in the formation of adenylosuccinate as a primary substrate for adenosine monophosphate (AMP) biosynthesis (Fig 2). Further, the expression of 5’-nucleotidase (Fig 2I), leading to the synthesis of adenosine from AMP, was higher than the expression of adenosine deaminase (Fig 2J), which plays a role in inosine formation, with logFC 2.41 and logFC 2.12, respectively. This finding indicates that cordycepin synthesis and adenosine biosynthesis result from a de novo pathway. Further, this result also suggests that the purine nucleotide in *C*. *militaris* is crucial for cordycepin production.

**Table 3:** Differentially expressed genes in purine nucleotide biosynthesis and related pathways of LSC and SMC of *C*. *militaris*.

Trinity transcript ID	NCBI-nr top hit	KEGG annotation	LogFC	LogCPM	FDR	Up/ Down
DN11023_c1_g1	SAICAR synthase [*C*. *militaris* CM01, XP_006669648]	Purine metabolism	6.6523	6.2860	9.88×10^−16^	Up
DN10264_c0_g1	Adenylos-succinate synthetase [*C*. *militaris* CM01, XP_006672557]	Purine metabolism	9.3833	4.2559	8.64×10^−9^	Up
DN9132_c0_g1	Glutamate synthase precursor [*C*. *militaris* CM01, XP_006668709]	Alanine, aspartate and glutamate metabolism	2.685	9.2645	5.58×10^−8^	Up
DN3696_c0_g1	NADP-specific glutamate dehydrogenase [*C*. *militaris* CM01, XP_006666603]	N/A	5.082	4.0156	1.05×10^−6^	Up
DN1703_c0_g1	5'-Nucleotidase [*C*. *militaris* CM01, XP_006665845]	N/A	2.4135	5.6876	5.40×10^−5^	Up
DN11002_c0_g1	Bifunctional purine biosynthesis protein [*C*. *militaris* CM01, XP_006670750]	Purine metabolism	1.9623	9.1166	7.29×10^−3^	Up
DN3243_c1_g1	Adenosine deaminase family protein [*C*. *militaris* CM01, XP_006672370]	Purine metabolism	2.1201	6.6737	1.19×10^−3^	Up
DN2159_c2_g1	Succinate-semialdehyde dehydrogenase [*C*. *militaris* CM01, XP_006670932]	Alanine, aspartate and glutamate metabolism	2.165	6.4537	6.51×10^−3^	Up
DN4124_c0_g2	Aspartate aminotransferase, putative [*C*. *militaris* CM01, XP_006668942]	Alanine, aspartate and glutamate metabolism	1.8127	5.4415	1.59×10^−2^	Up
DN3520_c0_g1	DNA-directed RNA polymerase I 135 kDa polypeptide [*C*. *militaris* CM01, XP_006673948]	Purine metabolism	-6.708	1.8949	2.29×10^−2^	Down

Expression of NADP^+^-specific glutamate dehydrogenase (DN3696_c0_g1, Fig 2A), GABA shunt, a glutamate synthase precursor (DN9132_c0_g1, Fig 2B) and succinate-semialdehyde dehydrogenase (DN2159_c2_g1, Fig 2D) were also upregulated (Table 4). Glutamate synthetase (Fig 2B) catalyzes glutamate formation from α-ketoglutarate and is further transferred to succinate by succinate-semialdehyde dehydrogenase via GABA pathway, which bypassed two steps in the tricarboxylic acid (TCA) cycle (Fig 2). Glutamine produced from glutamate can be converted to aspartate via glutamate by glutamate synthase, and glutamate to oxaloacetate by trans-aminated activity of aspartate aminotransferase (Fig 2C). Further, glutamate will be used for nucleoside biosynthesis and the aspartate also might role as an important precursor for SAICAR synthase and adenylosuccinate synthetase in cordycepin biosynthesis. This indicates that the expression of these genes is only induced under hypoxic conditions in the LSC.

**Table 4:** Differentially expressed genes related to the γ-aminobutyric acid (GABA) shunt, pentose phosphate pathway (PPP), glycolysis, and tricarboxylic acid cycle (TCA).

Trinity transcript ID	NCBI-nr top hit	logFC	logCPM	FDR	Up /Down
***GABA shunt***					
DN3696_c0_g1	NADP-specific glutamate dehydrogenase [*C*. *militaris* CM01, XP_006666603]	5.0824	4.0156	1.05 ×10^−6^	Up
DN2159_c2_g1	Succinate-semialdehyde dehydrogenase [*C*. *militaris* CM01, XP_006670932]	2.1648	6.4537	6.51 ×10^−3^	Up
DN9132_c0_g1	Glutamate synthase precursor [*C*. *militaris* CM01, XP_006668709]	2.6849	9.2645	5.58 ×10^−8^	Up
***PPP***					
DN5044_c0_g3	6-Phosphogluconate dehydrogenase [*C*. *militaris* CM01, XP_006672919]	-3.6160	4.1149	1.56 ×10^−3^	Down
DN10218_c0_g1	Aldolase [*C*. *militaris* CM01, XP_006665363]	2.8876	4.6909	3.24 ×10^−2^	Up
DN4197_c0_g1	Transaldolase [*C*. *militaris* CM01, XP_006674104]	2.0866	5.7998	1.38 ×10^−3^	Up
***TCA cycle***					
DN10291_c0_g1	Citrate synthase [*C*. *militaris* CM01, XP_006674075]	-1.3822	7.2302	3.66 ×10^−2^	Down
DN9520_c0_g1	Isocitrate dehydrogenase Idp1 [*C*. *militaris* CM01, XP_006673707]	-2.3592	8.4101	1.50 ×10^−5^	Down
DN7519_c0_g1	Succinate dehydrogenase iron-sulfur protein [*C*. *militaris* CM01, XP_006672347]	-1.3460	7.3435	4.0 ×10^−2^	Down

Furthermore, many genes that encoded a protein kinase and a member of the cytochrome P450 family, in total, 35 protein kinases and 20 cytochrome P450s, were upregulated in the LSC (S3 Table). Protein kinases catalyze the phosphorylation of proteins, which are involved in the regulation of various metabolism and gene expression pathways.

### Semi-quantitative RT-PCR (qRT-PCR)

**Fig 3:**
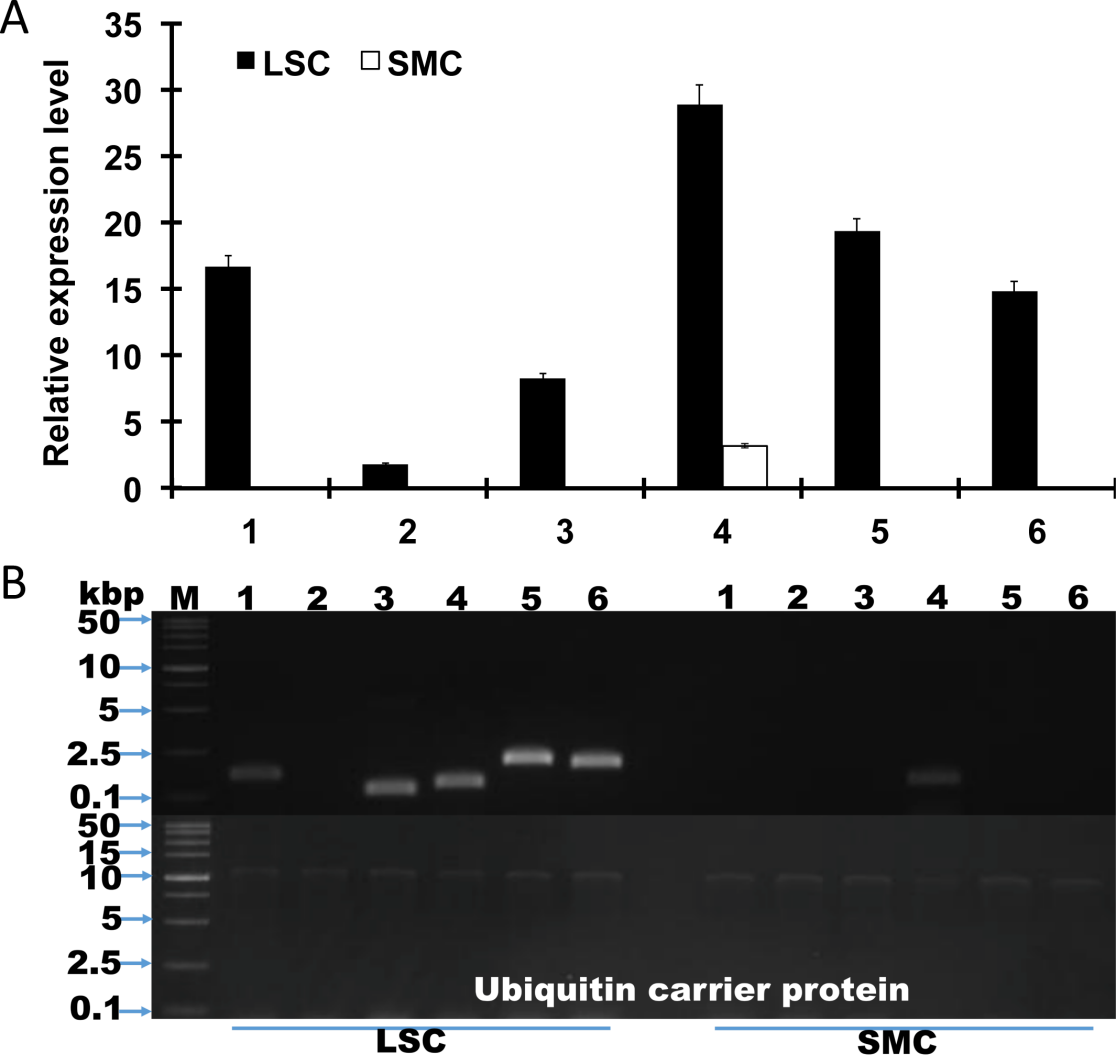
Semi qRT-PCR of each unigene in the LSC and SMC of *C*. *militaris*. RNA was extracted from mycelia cultivated for 5 d and 13 d in the LSC and the SMC, respectively. The ubiquitin carrier protein gene was used for the normalization of each gene expression. 1: Adenylosuccinate synthase; 2: Adenylosuccinate lyase; 3: Adenosine deaminase; 4: SAICAR synthase; 5: 5’-Nucleotidase; 6: IMP cyclohydrolase. Bars represent means ± 95% confidence intervals.

SemiqRT-PCR was conducted to validate the results of the RNA-Seq experiment. We used the ubiquitin carrier protein gene as a reference gene for this semi qRT-PCR experiment because the expression level of the ubiquitin carrier protein gene in the RNA-seq experiment was constant for both the LSC and the SMC (logFC 0.33; FDR 0.802) (S4 Table). Expression of SAICAR synthase, adenylosuccinate synthetase, 5’-nucleotidase, SAICAR synthase, the bifunctional purine biosynthesis protein, and adenosine deaminase family protein was significantly upregulated in the LSC compared with the SMC (Fig 3), and the optimal cycle number was 38, with a range of 23 to 43 cycles (S4 Fig). Both RNA-seq and semi-quantitative RT-PCR support their conclusions. In this experiment, the adenylosuccinate lyase gene was not amplified, even when its indifferent expression was confirmed in the RNA-seq experiment.

### Effect of aspartate addition to the culture on cordycepin production

**Fig 4:**
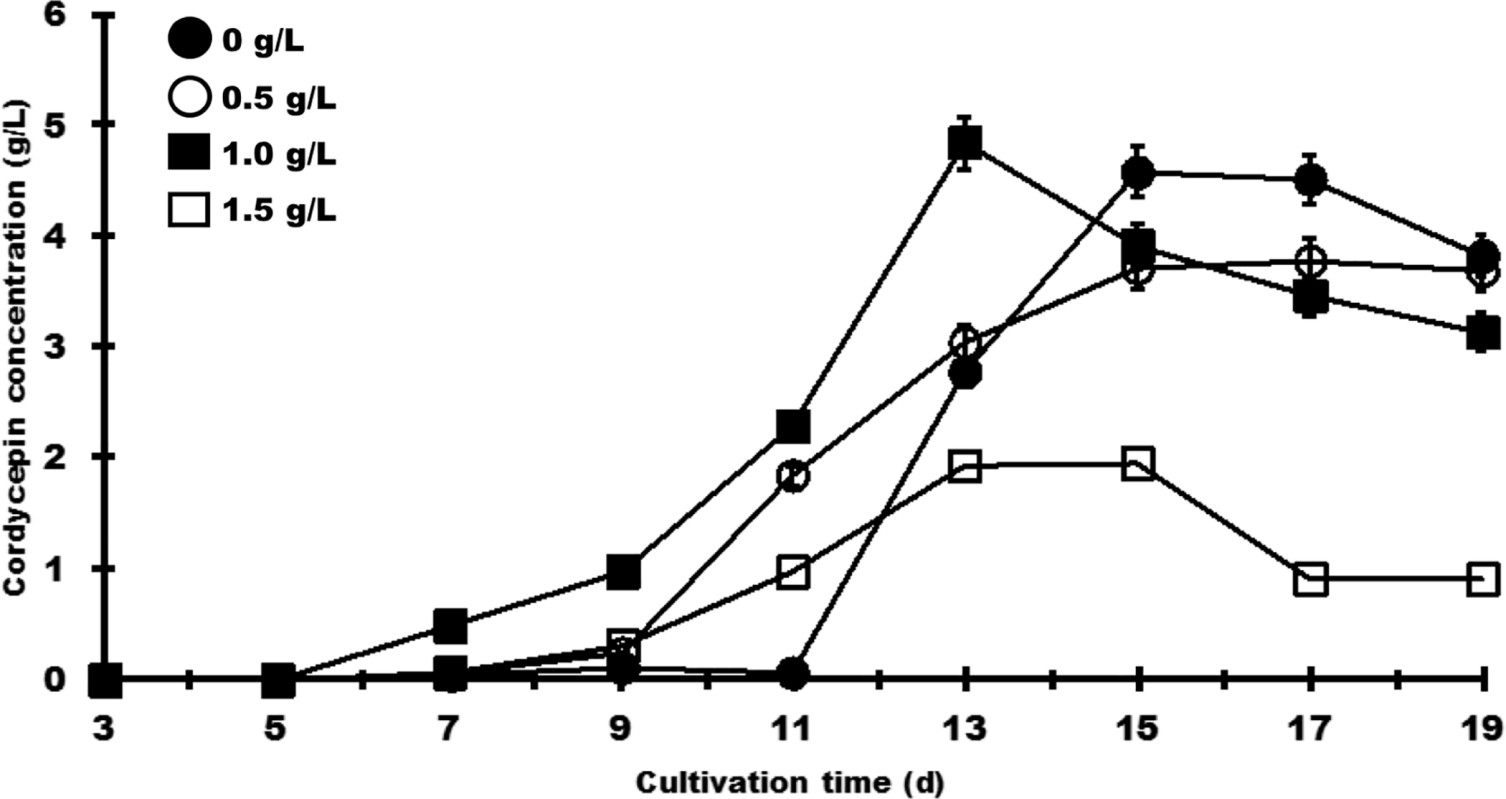
Effect of aspartate on cordycepin production in the LSC of *C*. *militaris* NBRC103752. Aspartate at each concentration was added to the culture medium, and the cultures were carried out for 19 d. The cordycepin concentration was measured every two days from 3 d to 19 d. The added aspartate concentrations were 0 g/L (closed circles), 0.5 g/L (open circles), 1.0 g/L (closed squares), and 1.5 g/L (open squares). The values are the results of the three technical repetitions (mean ± SD).

The RNA-seq experiment revealed that the expression of SAICAR synthase and adenylosuccinate-synthetase was greatly upregulated in the LSC. SAICAR synthase catalyzes the synthesis of SAICAR from CAIR. Adenylosuccinate synthetase leads to the synthesis of adenylosuccinate from IMP. These reactions require adenosine triphosphate (ATP), guanosine triphosphate, and aspartate. Our data also showed that aspartate aminotransferase (DN4124_c0_g2, Fig 2C), which catalyzes the reversible transfer of the amine group from L-aspartate to 2-oxoglutarate, was also upregulated by 1.8-fold in the LSC (Fig 2). This suggested that aspartate may be an important precursor for cordycepin production in *C*. *militaris*. When an aspartate concentration of 1 g/L was added, cordycepin production increased by 1.8-fold on the 13^th^ d and subsequently decreased (Fig 4). On the other hand, the addition of an amount of aspartate greater than 1.5 g/L reduced cordycepin production.

## Discussion

Cordycepin production is generally carried out by three culture methods, solid culture, SMC, and LSC. SMC is generally employed in industrial factories because of its short cultivation time, low chance of contamination, and high mycelial production and pellet formation in a compact space [31]. The present report emphasizes that the latter is not true as the mycelium is not formed in the SMC; instead, yeast-like is formed. This morphology was present as a consequence of agitation, which causes the hyphae to break. LSC is an alternative culture method for mycelial growth and secondary metabolite production [32].

In this study, more cordycepin was produced in LSC than in SMC of *C*. *militaris*. In SMC, cordycepin production began on the 4^th^ d of culture and a low concentration of 1 mg/L was attained by the 7^th^ d of culture, which subsequently decreased rapidly until the end of culture (inset of Fig 1). In the LSC, cordycepin decreased slightly on the 19^th^ d of culture, a trend that has been shown in previous reports [4–8; 15]. These phenomena suggest that feedback inhibition occurred, even though the exact reason has not yet been clarified.

In the LSC, thick mycelia covered the surface of the medium throughout the culture. This condition might be altered if the culture broth environment becomes hypoxic. It has been reported that in some filamentous fungi, gene expression in the GABA shunt and pentose phosphate pathways was upregulated during hypoxia [33–34]. Alternatively, under hypoxic conditions, these fungi downregulated the expression of some genes that are involved in gluconeogenesis and the TCA cycle.

Induction of cordycepin production in LSC in hypoxic medium was in line with a previous study [6], which showed that the dissolved oxygen level plays a major role in cordycepin biosynthesis as a high level of dissolved oxygen led to decreased cordycepin production. Further, GO enrichment analysis showed that “heme binding” and “iron ion binding” were significantly enriched in LSC of *C*. *militaris*, which led to cordycepin production (Table 2). It has been deduced that hypoxia and iron homeostasis are linked in some pathogenic fungi and that the expression of genes involved in heme biosynthesis is induced under hypoxic conditions [35–36]. The addition of ammonium (NH4^+^) to increased cordycepin production was clarified by the upregulation of NADP-specific glutamate dehydrogenase, aspartate aminotransferase and glutamate synthase [37–38]. It suggests involved in ammonia assimilation to maintain the nitrogen state and attributed to cordycepin production. Glutamine synthesizes from amination of glutamate and can be converted back to glutamate by glutamate synthase to balance the oxaloacetate level in TCA cycle since the high expression of NADP-specific glutamate dehydrogenase to produces glutamate.

Adenylosuccinate synthetase catalyzes the conversion of AMP to adenylosuccinate with aspartate, and it also has an allosteric activity that controls the AMP level. The gene encoding adenylosuccinate synthetase (DN10264_c0_g1) was highly expressed in the LSC of *C*. *militaris* compared with the SMC (Table 3). Supplementation with ferrous sulfate in the LSC led to a change in the expression level of adenylosuccinate synthetase and enhanced the production of cordycepin in *C*. *militaris* [39]. The *Cmwc-1* gene, which encodes a blue-light receptor, was disrupted in the *C*. *militaris* mutant that produced less cordycepin than wild-type *C*. *militaris*, and adenylosuccinate synthetase (CCM_07353, XP_006672557) was downregulated by 3-fold in the mutant compared with the wild-type [40].

An accumulation of SAICAR by upregulation of SAICAR synthase (DN11023_c1_g1) might trigger activation of the genes responsible for cordycepin biosynthesis in the LSC. It was previously found that small molecules, such as 5’-phosphoribosyl-5-amino-4-imidazole carboxamide (AICAR) and SAICAR, are involved in the transcriptional regulation of the purine biosynthesis pathway [41]. The transcriptome results showed that the expression level of adenylosuccinate lyase, which is a catalyst for AICAR transformation from SAICAR, was not significantly different between LSC and SMC. Overall our data support the importance of SAICAR synthase and Adenylosuccinate synthetase in cordycepin biosynthesis of *C*. *militaris*.

## Conclusion

In this study, a RNA-Seq experiment was conducted to investigate the differential expression of genes in the LSC and the SMC of *C*. *militaris*. The genes encoding SAICAR synthase and adenylosuccinate synthetase were greatly upregulated in LSC compared with SMC, suggesting that the purine nucleotide is crucial for cordycepin production. GABA shunt, NADH^+^-specific glutamate dehydrogenase and succinate-semialdehyde were upregulated, indicating that LSC induced a hypoxic condition during cultivation. Addition of aspartate enhanced the production of cordycepin in LSC. RNA-Seq analysis provided a comprehensive overview of the cordycepin biosynthesis pathway in *C*. *militaris*. Our study paves the way for unraveling the cordycepin biosynthesis pathway and improving cordycepin production in *C*. *militaris*.

## Supporting information

S1 Fig *C*. *militaris* NBRC 103752 morphology in LSC and SMC. Thick mycelia and only thin hypha and yeast like formed in LSC and SMC, respectively.
(TIFF)

S2 Fig Assessment for correlation of transcript counts among replicates of the LSC and SMC. (A) Multidimensional scaling (MDS) plot for transcript counts. The LSC samples were separated along dimensional 1 (x-axis). (B) Cluster dendrogram for log2 counts per million mapped reads (logCPM). Replicates of the LSC and the SMC divided into each cluster.
(TIF)

S3 Fig MA plot showing relationship between average logCPM and log2 fold-change (logFC). Red dots indicate differentially expressed genes at FDR <0.05 and horizontal blue lines indicate 2-fold changes.
(TIF)

S4 Fig Semi qRT-PCR of each unigene in the LSC and SMC of *C*. *militaris*. RNA was extracted from mycelia cultivated for the culture time of 5 d and 13 d in the LSC and the SMC, respectively. Ubiquitin carrier protein gene was used for the normalization of each gene expression.
(TIF)

S1 Note The edgeR script used for assessment of the transcript counts and identification of the differentially expressed genes. (TXT)

S1 Table Protein family and GO annotation. (XLSX)

S2 Table KEGG annotation and KO description. (XLSX)

S3 Table DEGs of protein kinase and cytochrome p450. (XLSX)

S4 Table DEGs of LSC and SMC. (XLSX)
