# Supplementary figures and images for: Insight into cordycepin biosynthesis of Cordyceps militaris: Comparison between a liquid surface culture and a submerged culture through transcriptomic analysis
Source: PLoS One. 2017 Nov 1;12(11):e0187052. doi: 10.1371/journal.pone.0187052 (PMC5665525; doi:10.1371/journal.pone.0187052)

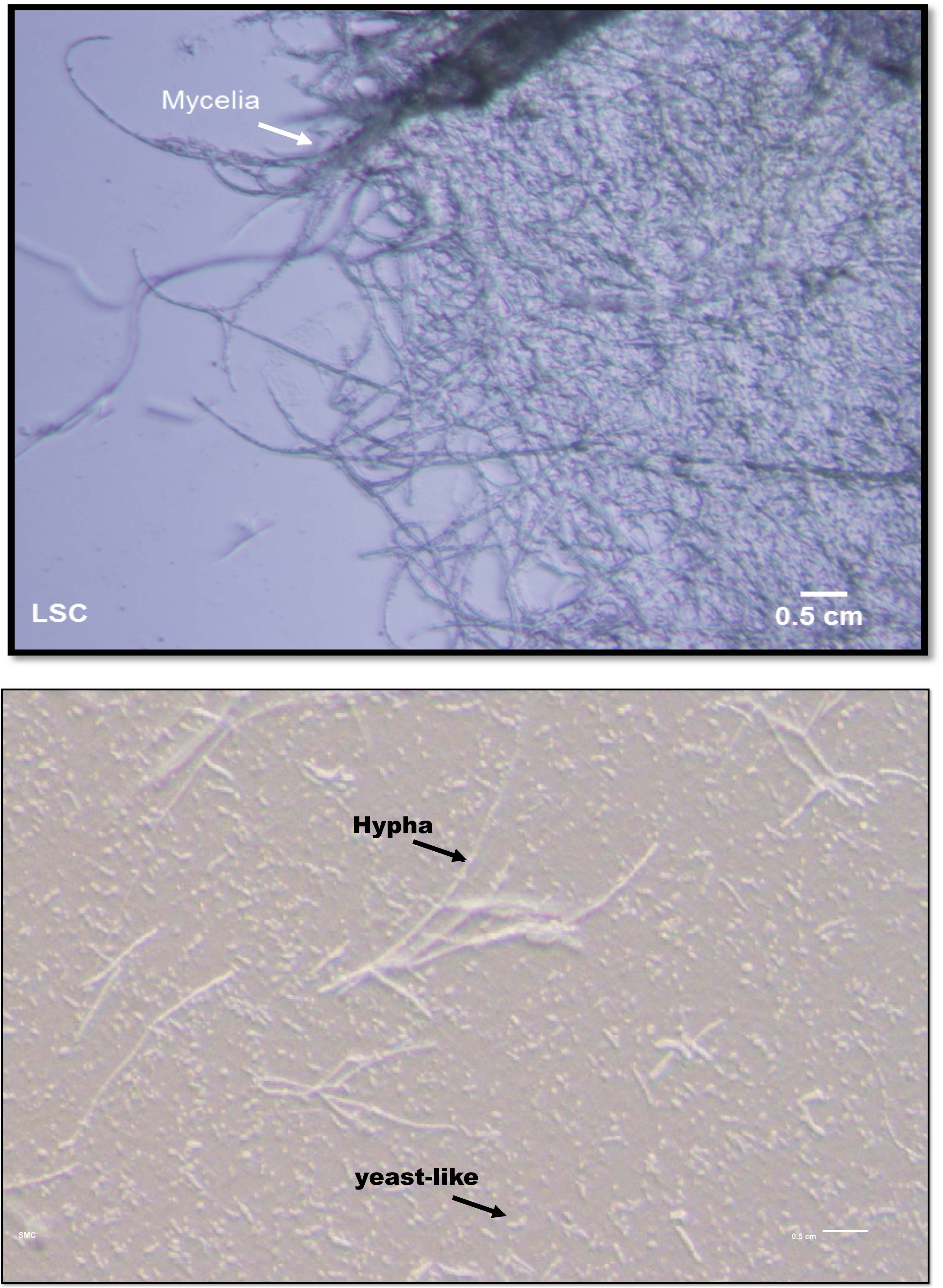

Supplement: S1 Fig — Thick mycelia and only thin hypha and yeast like formed in LSC and SMC, respectively. (TIFF) [file pone.0187052.s001.tiff]

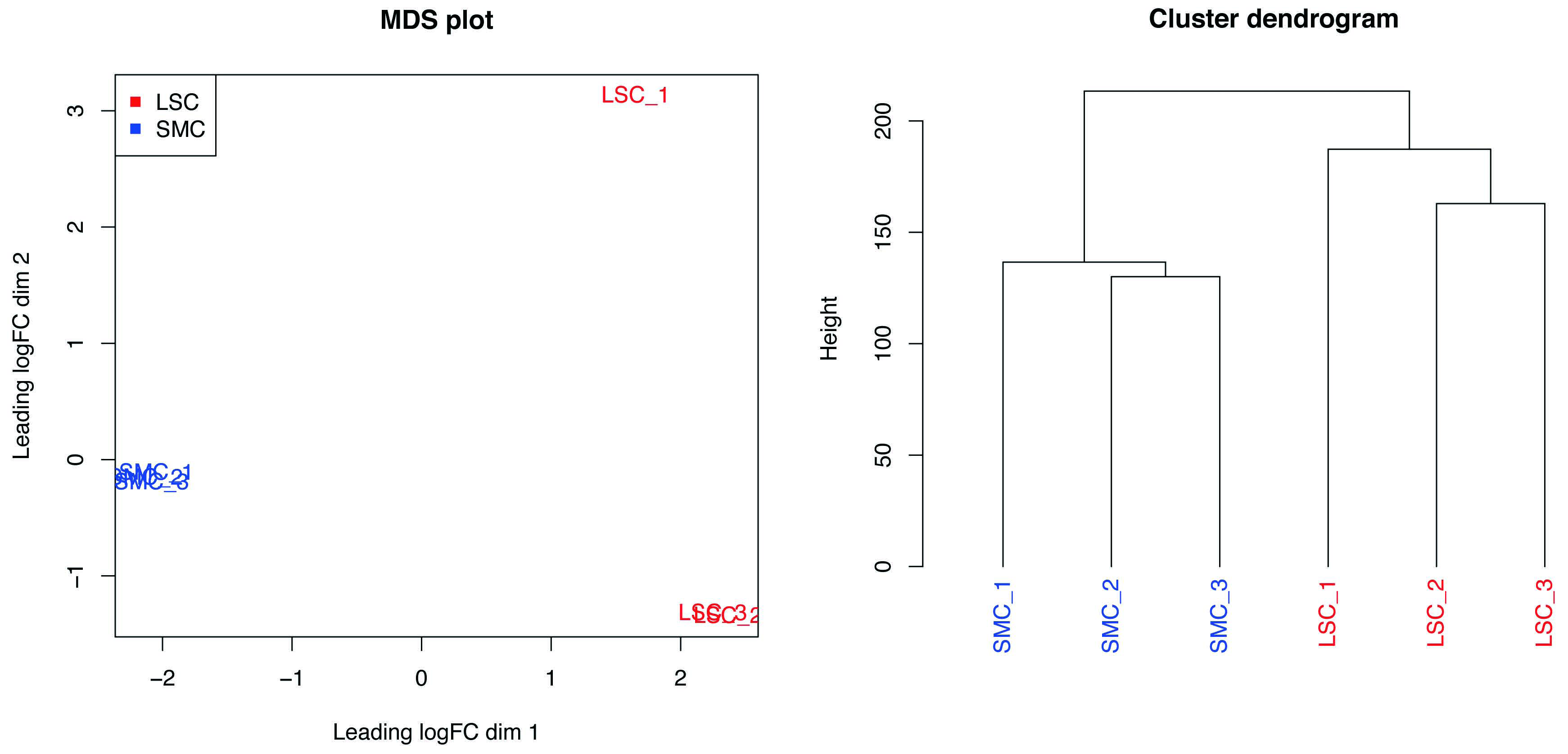

Supplement: S2 Fig — (A) Multidimensional scaling (MDS) plot for transcript counts. The LSC samples were separated along dimensional 1 (x-axis). (B) Cluster dendrogram for log2 counts per million mapped reads (logCPM). Replicates of the LSC and the SMC divided into each cluster. (TIF) [file pone.0187052.s002.tif]

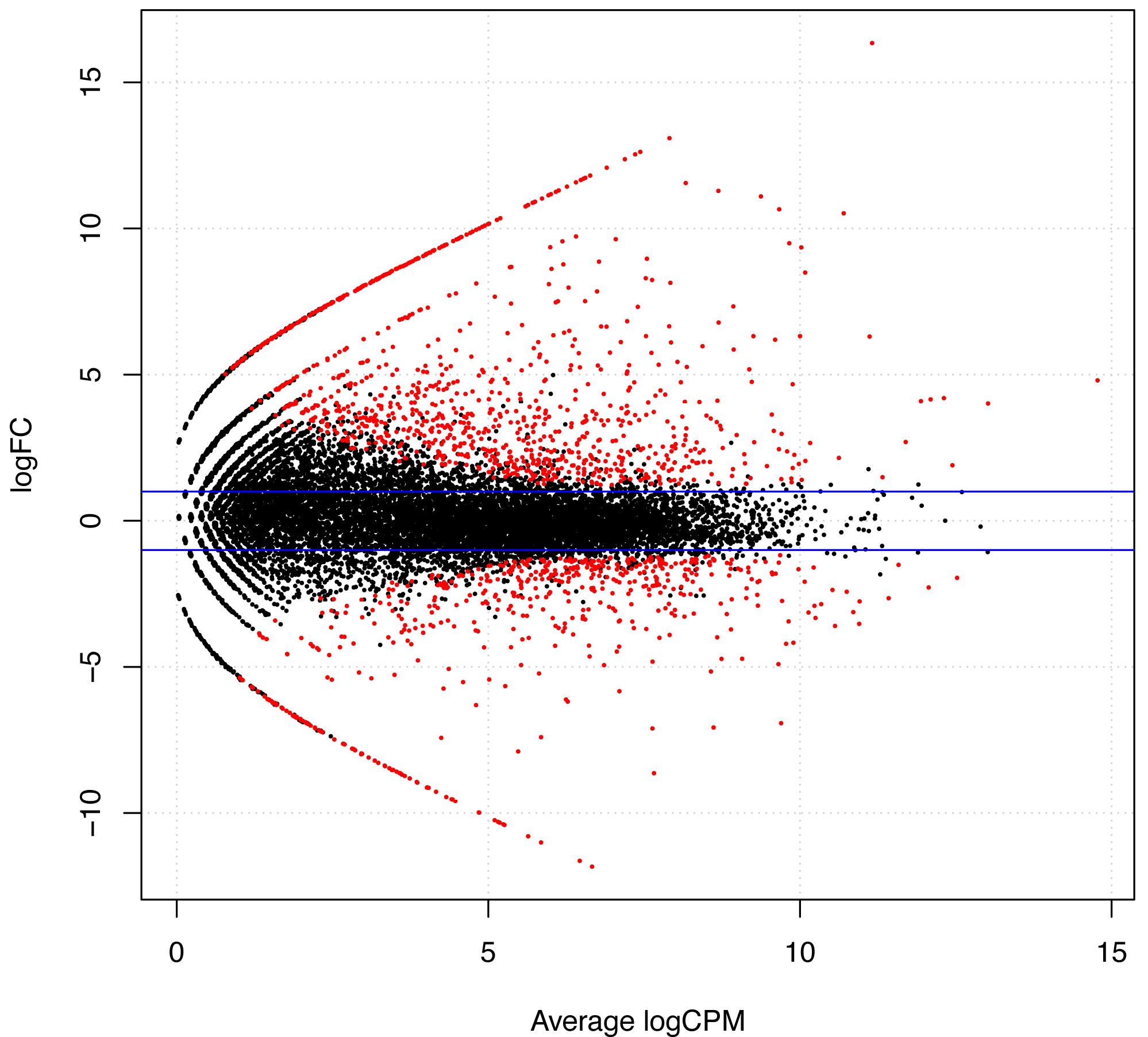

Supplement: S3 Fig — Red dots indicate differentially expressed genes at FDR <0.05 and horizontal blue lines indicate 2-fold changes. (TIF) [file pone.0187052.s003.tif]

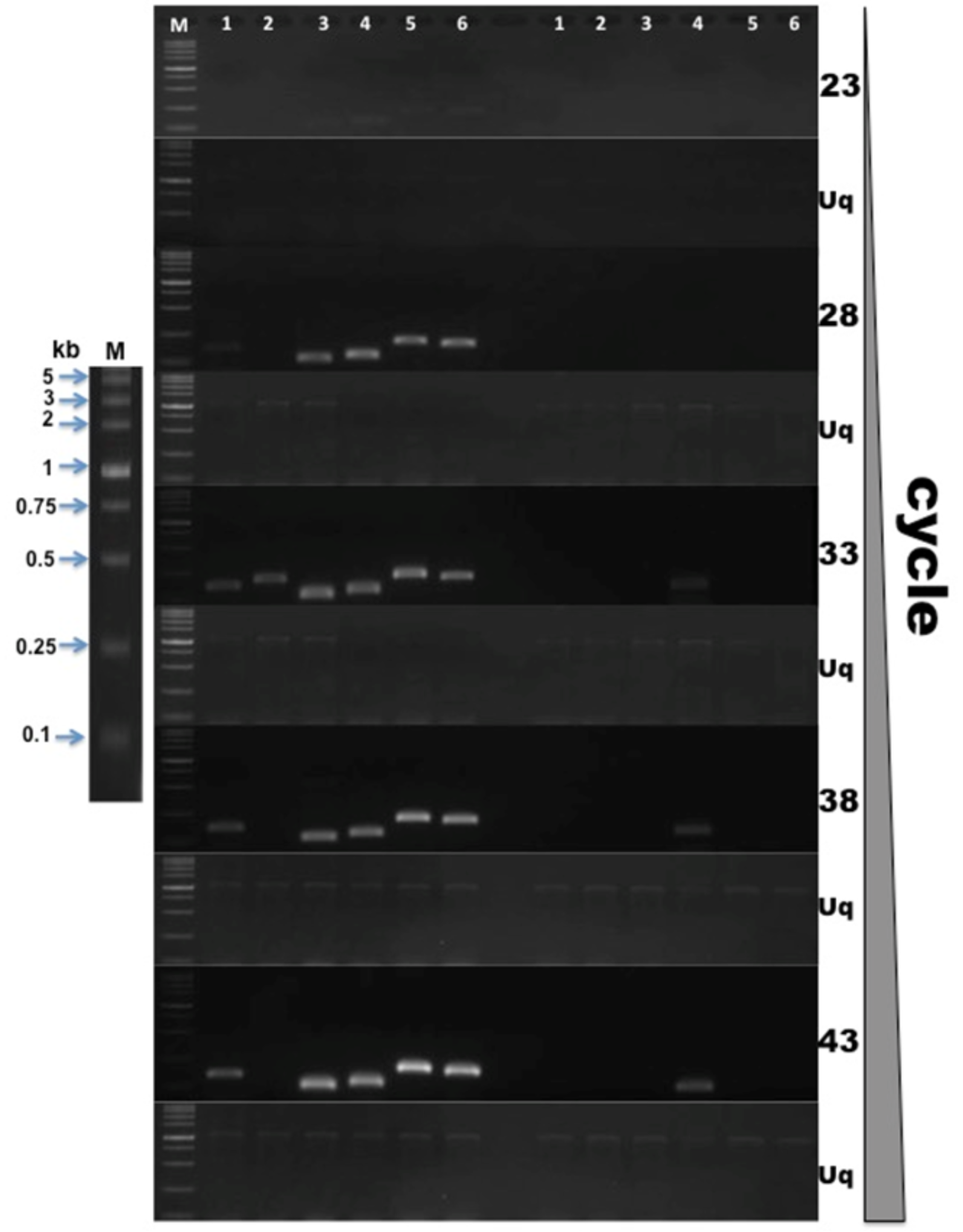

Supplement: S4 Fig — RNA was extracted from mycelia cultivated for the culture time of 5 d and 13 d in the LSC and the SMC, respectively. Ubiquitin carrier protein gene was used for the normalization of each gene expression. (TIF) [file pone.0187052.s004.tif]
